# A feedback regulatory loop involving p53/miR-200 and growth hormone endocrine axis controls embryo size of zebrafish

**DOI:** 10.1038/srep15906

**Published:** 2015-10-28

**Authors:** Jing Jing, Shuting Xiong, Zhi Li, Junjie Wu, Li Zhou, Jian-Fang Gui, Jie Mei

**Affiliations:** 1 College of Fisheries, Key Laboratory of Freshwater Animal Breeding, Ministry of Agriculture, Freshwater Aquaculture Collaborative Innovation Center of Hubei Province, Huazhong Agricultural University, Wuhan, 430070, China; 2 State Key Laboratory of Freshwater Ecology and Biotechnology, Institute of Hydrobiology, Chinese Academy of Sciences, University of the Chinese Academy of Sciences, Wuhan 430072, China

## Abstract

In vertebrates, growth hormone/insulin-like growth factor (GH/IGF) axis signaling plays a critical role in regulating somatic growth. Understanding the direct upstream regulators of GH/IGF axis remains a major challenge. Our studies of the zebrafish reveal that the conserved miR-200 family members are critical regulators of embryo size by targeting several GH/IGF axis genes, including *GH*, *GHRa*, *GHRb* and *IGF2a*. Overexpression of miR-200s led to cell cycle arrest in the G1 phase and induced apoptotic responses during embryo development, thereby inhibiting somatic growth of zebrafish embryos. Intriguingly, GH induced expression of both p53 and miR-200s, and miR-200s is a potential p53 transcriptional target, thus forming a negative feedback loop. Significantly, the up-regulation of miR-200s associated with GH activation is abolished in embryos with p53 mutation. By integrating these studies, we conclude that p53/miR-200 and GH/IGF signaling pathway form a negative regulatory loop to control embryo size, that provide critical insights into the long-standing puzzle of how body growth is determined during early development of teleosts.

In vertebrates, increasing evidences suggest that somatic growths during early embryonic and postnatal growth are greatly regulated by multiple endocrine systems including GH/IGF (growth hormone/insulin-like growth factor) axis^1^,^2^, CRH/POMC (corticotropin-releasing hormone/proopiomelanocortin) and melanocortin systems^3^. Growth hormone is a pituitary hormone that participates in numerous physiological processes including somatic growth and energy metabolism^4^. The elevated expressions of GH/IGF genes have been shown to be positively correlated with faster growth rate in mammalians^5^ and aquaculture fish species^6^,^7^,^8^,^9^. Moreover, GH transgenic mouse^5^ and aquaculture fish species^10^,^11^,^12^ show much faster growth rate than the control group.

MicroRNAs (miRNAs) have been identified as important modulators of development, cell differentiation, cell cycle and apoptosis by simultaneously silencing hundreds of target genes^13^. The expression level of multiple miRNAs has been directly linked to body growth during early development by regulating somatic stem cell proliferation and differentiation^13^,^14^. MiR-124 positively controls embryonic and adult growth of mouse by regulating target genes including sox9, forced expression of which results in a large size of embryos^15^. *Bantam* miRNA promotes somatic growth of *Drosophila melanogaster* by repressing the synthesis of the steroid hormone ecdysone that inhibits body growth^16^.

The miR-200 family is highly conserved in vertebrates, with miR-8 being the sole homolog in *Drosophila melan*ogaster. In human and mouse, the miR-200 family consists of five members that exist in two gene clusters, with miR-200a/200b/429 and miR-200c/141 in two different chromosomes. The members of miR-200 family display a high degree of sequence homology with only one nucleotide difference in their seed sequences. MiR-200a/141 and miR-200b/200c/429 have the same seed sequence, respectively^17^,^18^. MiR-8 promotes body growth in *Drosophila* by targeting USH/FOG2 that inhibits PI3K activity, or u-shaped (USH) that regulates insulin signaling^19^,^20^. Moreover, miR-8 regulates reproductive processes and proper secretion of lipophorin and vitellogenin in the female mosquito^21^. The miR-200 miRNAs are widely expressed in vertebrate organs, such as pituitary gland, thyroid gland, pancreatic islets, testes, prostate gland, ovary, breast, and liver^22^. As a direct downstream of p53, miR-200 miRNAs have been known as inhibitors of the epithelial-to-mesenchymal transition and tumor suppressors^23^. However, the function of miR-200 in body growth has not been reported in vertebrates.

In zebrafish, GH or growth hormone receptor (GHR) accelerated growth in transgenic zebrafish^24^,^25^, while *gh* mutant zebrafish exhibits a severe defect in somatic growth and severe dwarfism^26^. However, the direct regulation of GH/IGF axis genes during early development is still unclear. During embryo development, miR-200 family members have already been shown to express in the olfactory epithelia, epidermis, taste buds, pronephric duct, and neuromasts during embryo development by whole mount *in situ* hybridization^27^,^28^,^29^ (http://www.yale.edu/giraldezlab/miRNA%20insitu/miRNA-Insitu.html). In the present study, we found that miR-200s control body size by coordinately regulating cell growth, proliferation, and apoptosis that was distinct from its function in *Drosophila*, thereby providing a negatively feedback loop between p53/miR-200 and GH/IGF axis.

## Results

### Characterization and expression pattern of miR-200 family members during zebrafish embryo development

**Figure 1:**
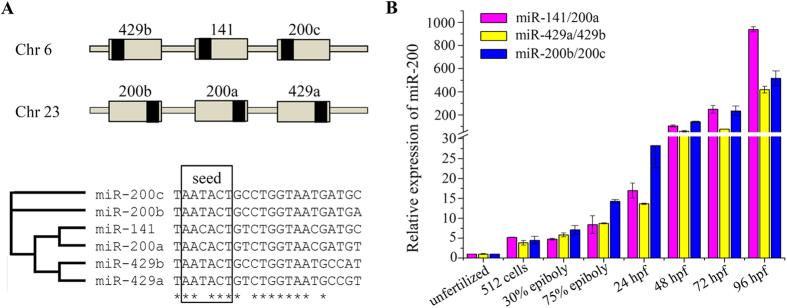
Genomic organization and expression pattern of zebrafish miR-200 family members. (**A**) Schematic illustration of genomic organization and multiple alignments of two miR-200 clusters. Grey boxes represent the pre-miRNAs and black boxes represent the mature miRNAs. (**B**) Expression of miR-200s during embryonic development. Error bars indicate mean ± SD, n = 3.

In zebrafish, the miR-200 family is composed of six family members (miR-200a, -200b, -200c, -141, -429a, -429b) clustered into two loci of chromosomes 6 and 23 (Fig. 1A). All the miR-200 family members were divided into three functional subgroups, miR-200b/200c, miR-200a/141 and miR-429a/429b, while miR-200b, -200c, -429a and -429b have the same seed sequence. Further, we performed RT-PCR to investigate the temporal expression pattern of miR-200s during embryo development (Fig. 1B). The expression of miR-200s was gradually increased after fertilization and kept at a high stable level from 48hpf to 96hpf during later embryogenesis.

### MiR-200s regulate the gene expression of growth hormone endocrine axes

**Figure 2:**
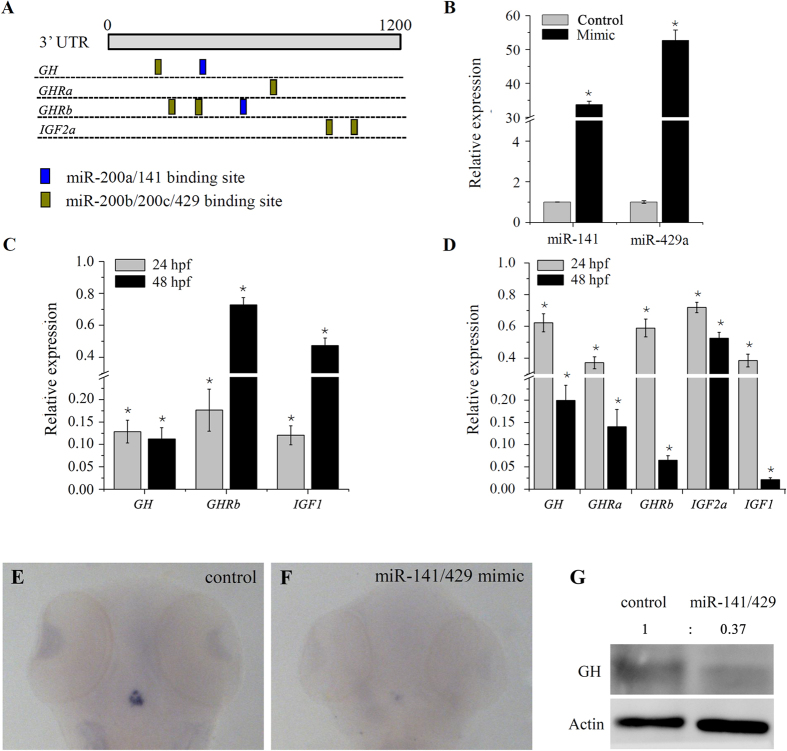
miR-200 s repress expression of multiple GH/IGF axis genes during embryo development. (**A**) Summary of the binding site of miR-200s in the GH/IGF axis genes predicted by Targetscan. (**B**) Expression of miR-141 and miR-429a in 24 hpf embryos injected with control or miRNA mimics. (**C,D**) Expression of GH/IGF axis genes in embryos injected with miR-141 and miR-429a mimic, respectively. (**E,F**) Whole mount *in situ* hybridization of *GH* in control and miR-141/429a mimic injected embryo at 48 hpf. (**G**) Protein expression of GH measured by western blot in 48 hpf embryo injected with miRNA mimics. (**B–D)** Error bars indicate mean ± SD, n = 3. Student’s t-test was used for statistical analysis (*p < 0.05).

Using the Targetscan bioinformatics algorithm, we found that several genes in the growth hormone endocrine axes, such as *growth hormone* (*GH*), *growth hormone receptor a* (*GHRa*), *growth hormone receptor b* (*GHRb*) and *insulin-like growth factor 2a* (*IGF2a*) were potential candidate targets of miR-200 family members (Fig. 2A and Fig. S1). MiRNA-target interactions have been shown to repress mRNA expression of most target genes^30^,^31^. So RT-PCR was performed to check the expression of these predicted target genes after ectopic expression of miR-200 family members. Injection of synthetic miR-141 and -429a mimic significantly increased the level of miR-141 and -429a transcript present (Fig. 2B). As the results, ectopic expression of miR-141 resulted in a reduction expression of *GH* and *GHRb* to 12.8% and 17.6% at 24 hpf, 11.2% and 66.4% at 48 hpf (Fig. 2C), while miR-429a reduced the expression of *GH*, *GHRa*, *GHRb* and *IGF2a* to 62.3%, 37.1%, 59%, 72% at 24 hpf and 20%, 14%, 6.5%, 52.5% at 48 hpf (Fig. 2D) compared to the control microRNA mimic. Moreover, *IGF1*, a major downstream mediator of the growth hormone pathway, was also significantly reduced by both miR-141 and miR-429a (Fig. 2C,D). For the following experiments, miR-141 and miR-429a mimics or inhibitors were 1:1 mixed to give working solutions. Further, whole-mount *in situ* hybridization at 48 hpf demonstrated a dramatic reduction of *GH* mRNA in pituitary following miR-141/429a injection (Fig. 2E,F). By Western blot analysis, we observed a reduction of GH protein in the zebrafish embryos subjected to miR-141/429a injection (Fig. 2G).

### MiR-200s regulate normal somatic growth of zebrafish embryo

**Figure 3:**
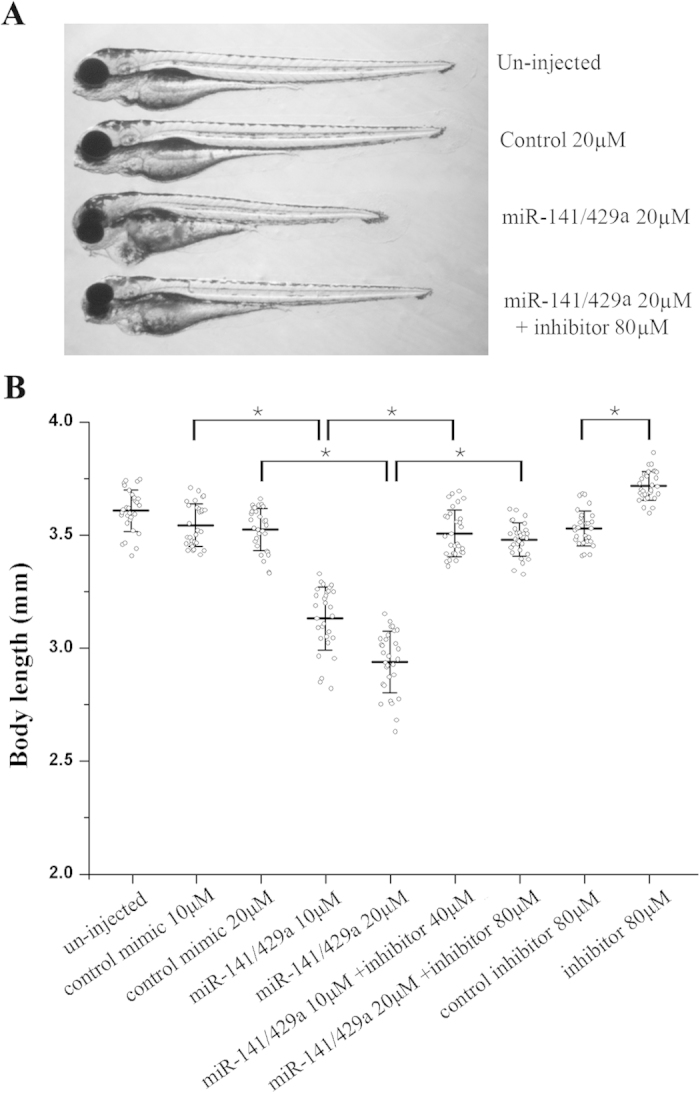
miR-200 s regulate somatic growth in zebrafish embryo. (**A**) Representative fish at 72hpf following injection with miRNA mimics and their inhibitors. (**B**) Body lengths (jaw to tail fin) of zebrafish embryos at 72hpf showed a dose-dependent suppression of somatic growth following ectopic expression of miR-141/429a mimics, and co-injection of miR-141/429a inhibitors partially rescued the growth defect. The indicated concentration of control mimic and inhibitor were used as control for the miR-141/429a mimics and inhibitors, and they have no obvious toxic effect on the embryo development. Error bars indicate mean ± SD, n = 30. Student’s t-test was used for statistical analysis (*p < 0.05).

Since miR-200s reduced the expression of *GH*, *GHRa*, *GHRb*, *IGF1* and *IGF2a* during embryo development, the effect of miR-200s on somatic growth was further investigated. At 72 hpf, over-expression of miR-141/429a dramatically reduced body length when compared to control mimic-injected embryos that have similar body length to the uninjected embryos. Ectopic expression of miR-141/429a led to pericardial edema in the embryos. Moreover, miR-141/429a inhibitors could rescue the defect of somatic growth resulted by miR-141/429a overexpression, whereas partially rescue the phenotype of pericardial edema (Fig. 3A). Dose-dependent suppression of somatic growth was clearly observed in miR-141/429a injected embryo at 72 hpf (Fig. 3B). An average body length of 3543 ± 94 μm (10 μM control miRNA mimic injection) and 3524 ± 94 μm (20 μM control miRNA mimic injection) were reduced to 3131 ± 140 μm (11.7% decrease in body length) and 2938 ± 136 μm (16.7% decrease in body length) by injection of 10 μM and 20 μM miR-141/429a, respectively (Fig. 3B). Then, we demonstrated the specific effect of miRNAs using a rescue experiment. Co-injection of either 10 μM miR-141/429a with 40 μM inhibitor or 20 μM miR-141/429a with 80 μM inhibitor produced a statistically significant recovery in length to 3507 ± 103 μm or 3480 ± 73 μm. Finally, an average length of 3529 ± 76 μm (80 μM control inhibitor injection) were elevated to 3717 ± 64 μm by injection of 80 μM miR-141/429a inhibitors, for a 5.3% increase in body length.

### Overexpression of miR-200s reduces cell proliferation and induces cell apoptosis during body growth of zebrafish

**Figure 4:**
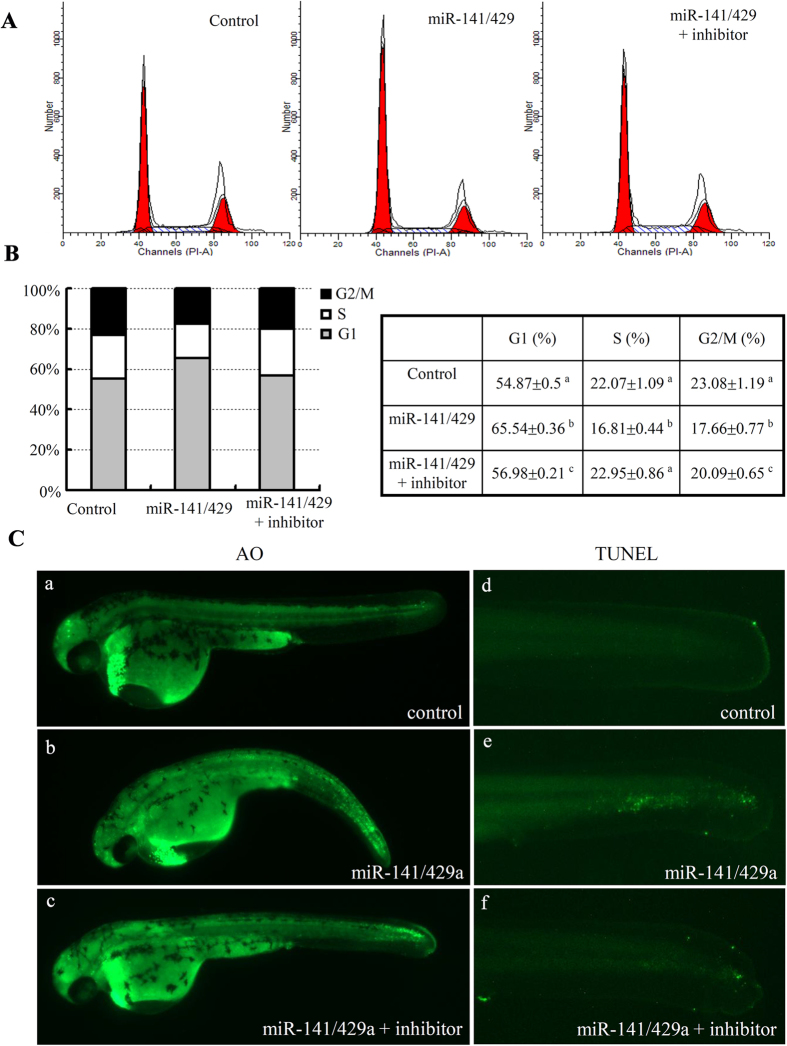
Analysis of cell proliferation and apoptosis phenotypes in zebrafish embryo with miR-200s overexpression. (**A**) Representative images of FACS analysis of DNA content in 48 hpf zebrafish embryo following injection with miRNA mimics and their inhibitors. (**B**) Graphical and tabular data for the percentage of cells population at G1, S, G2/M stages. Tukey’s test was used to compare the mean values among the experiment groups. Different letter indicated statistical significance. (**C**) The cell death phenotypes in whole embryos were revealed by AO staining (**a–c**) and apoptosis phenotypes in the tails were revealed by TUNEL assays (**d–f**), respectively.

Somatic cell proliferation and differentiation are usually involved in body growth during early development^13^,^14^. To determine the role of miR-200s in cell-cycle-progression of zebrafish embryos, FACS analysis was conducted to determine the DNA content of dissociated cells from miRNA mimic and control embryos at 24 hpf. In comparison to the control, overexpression of miR-141/429a increased percentage of cells in G1 (approximately 10.67%) and reduced percentages of cells in S (approximately 5.26%) and G2/M (about 5.42%) (Fig. 4A,B). Moreover, the miR-141/429a inhibitors could efficiently rescue the defects of cell cycle arrest. Accordingly, we used Acridine Orange to check status of cell death. Comparing with the control-mimic injected embryos (Fig. 4C-a), ectopic expression of miR-141/429a resulted in significantly higher level of apoptotic cells in brain and tails (Fig. 4C-b) that could be rescued by the miR-141/429a inhibitors (Fig. 4C-c). Furthermore, TUNEL staining was performed to confirm cell apoptosis in the tails of embryos. The cell apoptosis led by overexpression of miR-141/429a could be efficiently rescued by miR-141/429a inhibitors (Fig. 4C-d–f). Taken together, our observations suggest that miR-141/429a inhibits cell proliferation and induces cell apoptosis.

### Multiple critical factors of growth hormone endocrine axes are direct targets of miR-200s

**Figure 5:**
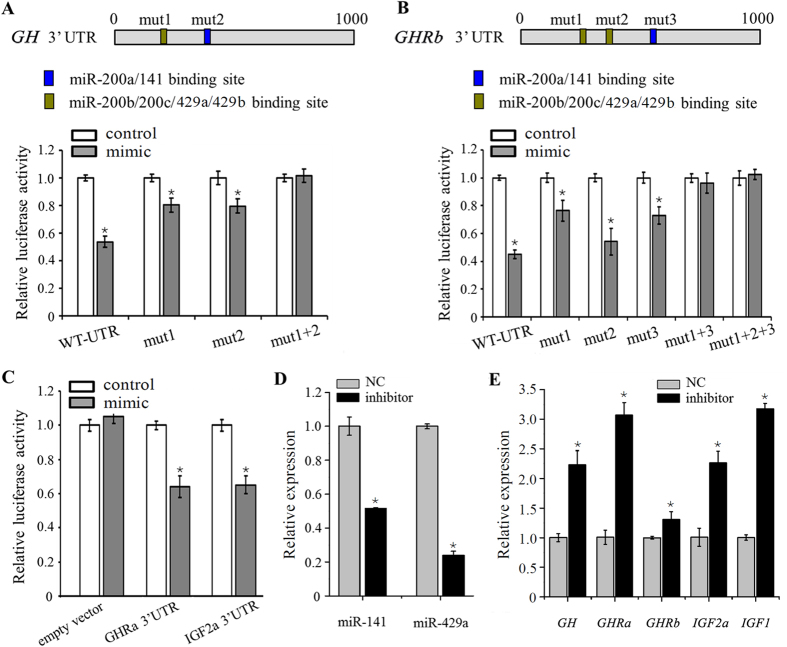
miR-200s directly target multiple GH/IGF axis genes. (**A–C**) Dual-luciferase reporter assay for validation of miR-200s target sites in the 3′ UTR of *GH*, *GHRb*, *GHRa* and *IGF2a*. Conserved miR-200–binding sites in *GH* and *GHRb* 3′ UTR are indicated and mutated separately. Luciferase assays were performed in triplicate and are representative of 2–3 independent experiments. (**D**) Levels of miR-141 and miR-429a at 24 hpf embryos injected with control or miRNA inhibitors. (**E**) Expression of GH/IGF axis genes in embryos following injection of miRNA inhibitors. (**A–E**) Error bars indicate mean ± SD, n = 3. Student’s t-test was used for statistical analysis (*p < 0.05).

To determine whether *GH*, *GHRa*, *GHRb* and *IGF2a* are direct target genes of zebrafish miR-200s, we firstly performed luciferase reporter assays by linking 3′ UTR of these putative target genes to the C-terminus of Firefly luciferase present in pmirGLO vector. miR-141/429a repressed the luciferase activity of *GH* 3′ UTR-pmirGLO, whereas mutation of either predicted miR-200a/141 or miR-200b/200c/429a/429b binding site attenuated this repression, and mutation in both binding sites abrogated this repression (Fig. 5A). Among all three binding sites of miR-200s in *GHRb* gene, mutation of either binding site 1 or 3 attenuated the repression of luciferase activity by miR-141/429a, and mutation in both binding sites 1 and 3 abrogated this repression (Fig. 5B). In addition, the luciferase activity of *GHRa* 3′ UTR-pmirGLO and *IGF2a* 3′ UTR-pmirGLO was also repressed by miR-429a compared with the control miRNA mimics (Fig. 5C).

To examine the ability of miR-200s inhibitors to repress miR-200s levels *in vivo*, we injected zebrafish embryos with anti-miR-141, anti-miR-429a or a scrambled control. The expression levels of miR-141 and -429a were efficiently reduced to 51.59% and 23.98% by inhibitors (Fig. 5D). Moreover, the mRNA levels of *GH*, *GHRa*, *GHRb*, *IGF2a* and IGF1 were significantly elevated when miR-141/429a was repressed (Fig. 5E). These results demonstrate that *GH*, *GHRa*, *GHRb* and *IGF2a* are direct target genes of zebrafish miR-200s.

### Growth hormone reciprocally regulates expression of miR-200s depending on p53

**Figure 6:**
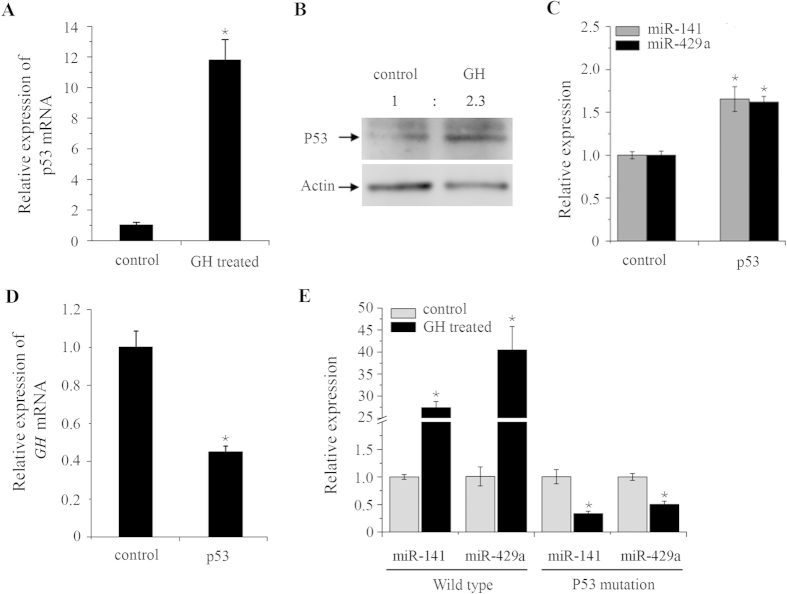
GH activates miR-200s expression in a p53-dependent manner. (**A**) Increased expression of p53 mRNA by GH overexpression. (**B**) Increased expression of p53 protein by GH overexpression. Numbers indicate quantification of the P53 band densities relative to actin. (**C**) Increased expression of miR-141 and miR-429 at 24 hpf embryos when injected with p53 mRNA. (**D**) Decreased expression of *GH* at 24 hpf embryos when injected with p53 mRNA. (**E**) The expression of miR-141/200a and miR-429a/b in wild-type and p53 mutation embryos when GH was overexpressed. Nanopure water injected embryos as a control. (**A**,**C–E**) Error bars indicate mean** **± SD, n = 3. Student’s t-test was used for statistical analysis (*p < 0.05).

Recent study suggested that GH is necessary for the increased adipose p53 expression in obese mice^32^. Therefore, we checked the mRNA and protein level of p53 in zebrafish embryos after injection of either recombinant human GH protein or zebrafish *GH* mRNA. Human GH induced expression of both p53 mRNA and protein (Fig. 6A,B). Similarly, ectopic expression of zebrafish *GH* resulted in upregulation of both *GH* and *p53* mRNA level (Fig. S2-A and S2-B). As a direct downstream of p53, miR-200 miRNAs have been known as inhibitors of tumor cell proliferation and growth^23^. Accordingly, we detected that expression of miR-141 and miR-429a were increased by injection of p53 mRNA into zebrafish embryos compared to the control groups (Fig. 6C), whereas the *GH* mRNA was reduced by ectopic expression of p53 (Fig. 6D). Given that GH activates p53 expression and p53 induced miR-200s expression, we suspected that GH may positively regulate miR-200s. Notably, both miR-141 and miR-429a were increased after GH injection (Fig. 6E). Moreover, the inductions of both miR-141 and miR-429a by GH injection were abrogated in p53 mutant embryos, suggesting that p53 is necessary for the increased miR-200s expression by GH activation in zebrafish embryos.

### The potential p53–binding sites in miR-200 promoters were responsible for promoter activity

**Figure 7:**
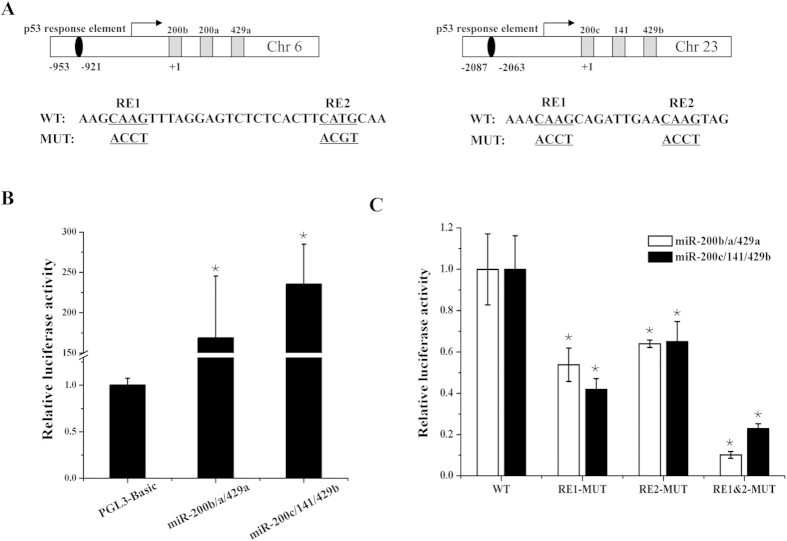
p53 contributes to the transcription of miR-200s. (**A**) A schematic depiction of the miR-200b/a/429a and miR-200c/141/429b promoter fragments. The sequence information of the putative p53 response element (RE) in wild types (WT) and their mutant (MUT) were indicated. (**B**) Analysis of the transcriptional activity using the promoters of miR-200s clusters by luciferase assay. (**C**) Luciferase assays of miR-200s cluster promoters (WT or MUT of p53 RE) co-transfected with pcDNA3.1 (+)-p53-ORF plasmid in HEK293T cells. The activity of miR-200s promoters were declined in mutant groups. (**B,C**) Error bars indicate mean ± SD, n = 3. Student’s t-test was used for statistical analysis (*p < 0.05).

In the promoters of zebrafish miR-200 clusters, two putative response elements (RE1 and RE2 as a half-site) were identified from miR-200b/a/429a and miR-200c/141/429b promoters, respectively^33^. Considering that p53 activates miR-200 promoter activity in human^23^, we evaluated the role of RE1 or RE2 for the transcriptional activity of promoters. We introduced a single mutation into RE1 or RE2 or simultaneous mutations into both RE1 and RE2 (Fig. 7A). Compared with the PGL3-Basic empty vector, the constructed PGL3-miR-200s promoter vectors showed higher luciferase activity (Fig. 7B), indicating the promoter fragments of miR-200s cluster have a promoter activity. As shown in Fig. 7C, mutation of either RE1 or RE2 led to a declined luciferase activity of miR-200b/a/429a and miR-200c/141/429b promoters. Further, there was a more declination of luciferase activity when both RE1 and RE2 were mutated. These results suggest that p53 response element in miR-200s cluster promoter play a critical role in transcriptional activation.

## Discussion

**Figure 8:**
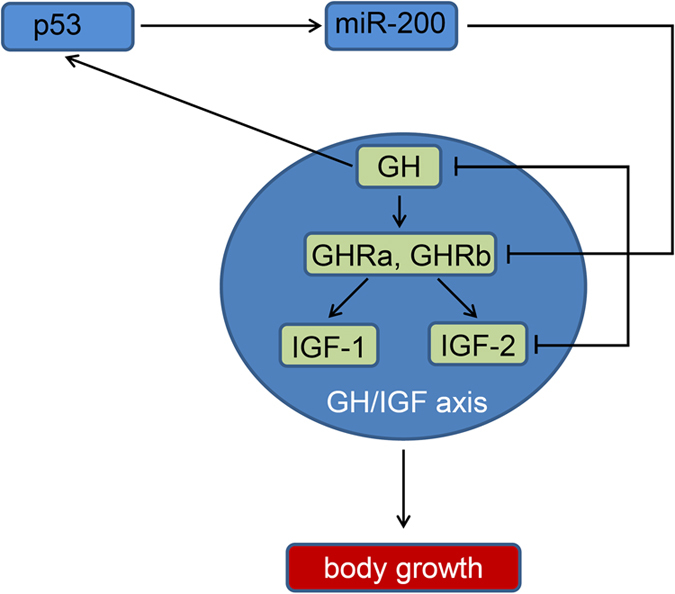
Model for the feedback regulatory loop involve p53/miR-200 and GH/IGF axis. In zebrafish embryo, miR-200 family members regulate body growth by directly repressing critical GH/IGF axis genes, *GH*, *GHRa*, *GHRb* and *IGF2a*. Further, p53 activity is induced by GH, thereby resulting in the up-regulation of miR-200s that are potential transcriptional target of p53, thus forming a negative feedback loop.

The results of this study reveal miR-200s as important regulators of somatic growth during embryo development. Overexpression of miR-200s in zebrafish embryo results in a decrease in body length and an apoptosis and cell cycle arrest phenotype. miR-200s directly repress *GH*, *GHRa*, *GHRb* and *IGF2a* mRNA such that knockdown of miR-200s causes increased expression of these genes. Reciprocally, GH promotes the expression of both p53 and miR-200s. Embryos lacking p53 impaired the activation of miR-200s by GH. A model to account for the role of miR-200s in mediating somatic growth and cell death during embryonic development is shown in Fig. 8. Our results provide insights into the roles of miRNAs as “fine-tuners” of embryonic growth under conditions of GH activation.

GH/IGF signaling system is a master regulator stimulating cellular and somatic growth in vertebrates. Mutation of either GH or IGF1 resulted in growth retardation of postnatal mice^2^ or led to dwarfism in the rat^34^. In zebrafish, ectopic expression of GH/IGF axis genes accelerated growth in transgenic zebrafish^24^,^25^, while *GH* mutant zebrafish exhibits a significant decrease of somatic growth and severe dwarfism^26^. Given this, it is not surprising that the embryonic growth is reduced (16.7% decrease) by miR-200s which target several important genes in the GH/IGF axis, such as *GH*, *GHRa*, *GHRb* and *IGF2a* (Figs 2 and 3). In contrast, embryos injected with miR-141/429a inhibitors showed only 5.3% increase in body length, because the expression change of GH/IGF axis genes was more remarkable in embryos treated with miR-141/429a mimics than with their inhibitors (Fig. 5E).

Ectopic expression of miR-141/429a mimics lead to pericardial edema in zebrafish embryos, which could be partially rescued by miR-141/429a inhibitors (Fig. 3A). However, injection of miR-141/429a inhibitors did not cause any observable developmental defects in zebrafish embryos, that was the same phenotype as injection of morpholinos of miR-200 family members^35^,^36^. The results in our study suggest that over-expression of miR-141/429a may affect embryo heart development. Serum response factor (SRF), whose 3′ UTR is directly targeted by miR-200b^37^, recruits myocardin to co-activate transcription of downstream cardiac gene^38^,^39^,^40^. GATA-binding protein 4 (Gata-4), a direct target of miR-200b, plays an important role in the processes of heart development^41^. The role of miR-200s in cardiac function and relevant downstream genes need to be further identified and studied. Apart from inhibiting body growth in embryo, overexpression of miR-141/429a resulted in cell cycle arrest and cell apoptosis (Fig. 4). And somatic cell proliferation and differentiation are usually involved in body growth during early development^13^,^14^. As a tumor suppressor, the miR-200 family has anti-growth and anti-differentiation function in cancer cells^41^,^42^.

MiR-8/200s activity in somatic growth was first discovered in *Drosophila* and characterized as a growth-promoting factor by promoting insulin and PI3K signaling^19^,^20^. However, miR-200s repress insulin signaling in zebrafish. As a conserved miRNA family, miR-8/200 family members display extensive functional divergence between invertebrates and fish species, which serve as an evolutionary link between invertebrates and higher vertebrates. In mammals, miR-141 and miR-200c co-existed in one gene cluster without miR-429, whereas miR-429b, another duplicated copy of miR-429 appears in zebrafish and co-existed with miR-141/200c in chromosome 6 (Fig. 1A). Besides regulating somatic growth, GH is also implicated in energy metabolism, gonadal development, osmoregulation and immunity in fish as well^4^,^43^. Moreover, miR-200s were revealed to regulate the response to osmotic stress and olfactory neurogenesis in zebrafish embryos^28^,^35^, and have potential roles for testis development in yellow catfish^44^. MiR-8 regulates reproductive processes in the female mosquito by targeting the Wingless signaling pathway^45^. Therefore, the evolutionary conservation of miR-8/200s implies that some important functions emerge after the appearance of fish species and even the colonization of aquatic environments. However, the functional relationship between miR-8/200s and GH is still not clear in these processes.

The expression of a subset of miRNAs appears to be transcriptionally regulated by p53 in response to various physiological stresses, including growth stress^46^,^47^,^48^. Despite reports demonstrating a direct transcriptional regulation of miR-200s by p53 in mammals, the role of p53 in somatic growth is unclear^23^. When coupled to our findings related to growth stress, a clear theme emerged is that a function of p53 is to regulate the response to growth stress induced by GH overexpression and then activate miR-200s which reciprocally target several important GH/IGF axis genes. In contrast to the severe developmental defects in the brain and craniofacial of p53 deficiency mice^49^, the embryonic development was normal in p53 mutant zebrafish^50^. In rat and human pituitary cells, there is a marked induction of intracellular pituitary GH after p53-mediated senescence, GH is a direct p53 transcriptional target and could not be induced in cells losing p53 function^51^. Here, we have identified p53 regulates GH expression through miR-200s in zebrafish. Through gain-of-function and rescue approaches, we provide a novel model of molecular interactions between p53/miR-200s and GH/IGF axis that is necessary for somatic growth during embryo development (Fig. 8).

## Materials and Methods

### Fish care

Experiments involving zebrafish were approved by the institution animal care and use committee of Huazhong Agricultural University and the methods were carried out in accordance with the approved guidelines. Wild-type AB line and Tp53 mutant zebrafish were maintained according to the established protocols and staged by morphology and age (hours post fertilization, hpf)^52^. Tp53 mutant zebrafish was obtained from Wuhan Xiao (Institute of hydrobiology, Chinese Academy of Sciences) and originally from A Thomas Look (Harvard Medical School)^50^,^53^.

### qRT-PCR analysis and whole mount RNA *in situ* hybridization

Total RNA was isolated from each sample with 30 embryos using miRNeasy Mini Kits (Qiagen) and subjected to DNase I treatment (Invitrogen). Oligo (dT) primer and stem-loop RT primers (Table S1) were used for cDNA synthesis using Superscript II reverse transcriptase (Invitrogen). There is only one nucleotide difference in stem-loop RT primer between miR-141 and miR-200a, miR-429a and miR-429b, miR-200b and miR-200c, respectively. To check the expression of miR-200 family members during normal embryonic development, we synthesized cDNA with a mixture of stem-loop RT primers of miR-141/200a, miR-429a/429b and miR-200b/200c, respectively. For other experiments, we synthesized cDNA with the specified stem-loop RT primer to of miR-141 or miR-429a. Then, quantitative RT-PCR (qRT-PCR) reactions were performed by using iTaq™ Universal SYBR Green Supermix (Bio-Rad) and run on the CFX96 Touch™ Real-Time PCR Detection System (Bio-Rad). Each experiment was performed in triplicate and the data was analyzed using the 2^–ΔΔCt^ program. The abundance of miRNA and mRNA was normalized to U6 snRNA and 18s rRNA, respectively. Primers are available in Table S2.

For whole mount *in situ* hybridization, probe preparation, probe hybridization and embryo staining was performed as described previously^54^.

### Microinjection and body length measurement

MicroRNA mimics and corresponding negative controls were synthesized by Genepharma (Shanghai, China) and microinjected at a concentration of 10 μM or 20 μM. For the rescue, miRNA inhibitors and corresponding negative controls were microinjected with mimics at a concentration of 40 μM or 80 μM. Zebrafish *GH* and *p53* ORF were subcloned into the pCS2 + vector for *in vitro* transcription. Capped sense RNAs were synthesized using SP6 RNA polymerase following the manufacturer’s instructions and injected at a concentration of 100 ng/μL. In addition, 10 pg recombinant human growth hormone (rhGH, ProSpec) and nanopure water were injected as GH treatment and control group, respectively. All the microinjection was conducted at single-cell zebrafish embryos with 1 nl injection volume.

Zebrafish embryos at 3dpf were anesthetized in tricaine methanesulfonate (MS222, Sigma) and imaged by Leica MZ16FA Microscope using MetaVue software. The body length (forehead to tail fin) was measured using ImageJ software (30 embryos measured per group).

### Flow cytometry analysis and apoptosis detection

Embryos (30 per group) injected with miRNA mimics were dissociated, PI stained and then sorted on a BD FACS Calibur flow cytometer as described previously^55^. The percentages of cell phases within the cell cycle were analyzed using Modfit software. Cell apoptosis in whole mount was detected by acridine orange (AO) staining and terminal transferase dUTP nick end labeling (TUNEL) assays as described previously^54^.

### Western blot

The embryos were manually de-yolked and lysed in the lysis buffer. Equal amount of protein samples were separated in 10% SDS-PAGE gels and transferred onto a nitrocellulose membrane using standard protocols. After blocking, the membranes were incubated with primary antibodies, rabbit polyclonal anti-p53 (GeneTex, GXT128135) or mouse monoclonal anti-GH (made and kindly provided by Dr. Wei Hu’s lab, Institute of Hydrobiology, Chinese Academy of Sciences) and anti-β-actin (Cell signaling, 4967S). The blot was detected with HRP-conjugated secondary antibodies and visualized using an enhanced chemiluminescence (ECL) detection reagents kit^56^.

### Luciferase reporter assay

3′UTR fragments of target genes (*GH*, *GHRa*, *GHRb*, *IGF2a*), which contain one or more putative miR-200 binding sites were inserted into the pmir-GLO plasmid (Promega). Then, the binding sites of miR-200a/141 (CAGTGTT) and miR-200b/200c/429 (CAGTATT) in the constructed wild-type plasmids were replaced with TGACGCG and TCAGTCG by site-directed mutagenesis^57^, respectively. For *GH*, mut1 and mut2 represented mutations of miR-200b/200c/429 and miR-200a/141 binding sites. Mut 1/mut 2 and mut 3 in *GHRb* 3′UTR correspondingly represented mutations of miR-200b/200c/429 and miR-200a/141 binding sites. And mut 1 + 2/mut 1 + 3 and mut 1 + 2 + 3 represented double and triple mutations for the miR-200 binding sites. HEK-293 T cell were transiently transfected with 25 ng plasmid (wild-type or mutant) and 50 nM miRNA mimics or negative control per 24-well using DharmaFECT transfection reagent (Dharmacon). Luciferase activity was measured at 24 h post transfection using Dual Luciferase reporter assay system (Promega). Relative reporter activities were determined by normalizing *Firefly* activity to *Renilla* activity.

Promoter sequences of miR-200b/-200a/-429a and miR-200c/-141/-429b were cloned from normal zebrafish genomic DNA and inserted into pGL3-basic plasmid (Promega) without an internal promoter element. The putative p53 response elements in promoter sequences were mutated to get series mutant promoter vectors. The ORF of zebrafish p53 was amplified and subcloned into pcDNA3.1 (+) vector (Invitrogen). 250 ng PGL3-miR-200 promoter plasmid or PGL3-Basic empty vector was transfected into HEK-293 T cell using 25 ng pRL-TK vector (Promega) as a control. Furthermore, various PLG3-miR-200 promoter construct vector (wild-type or mutant) was co-transfected with 500 ng pcDNA3.1 (+)-p53-ORF plasmid. After 24 h post transfection, the cells were collected and used for luciferase assay. At least three independent experiments were performed.

### Statistical analysis

Data was shown as mean ± SD. Significance of difference between two groups was analyzed by Student’s t-test. Tukey’s test was used to compare the mean values among the experiment groups. Statistical analysis was performed with SPSS software (SPSS Inc.). A probability (P) of <0.05 was considered statistically significant.

## Additional Information

**How to cite this article**: Jing, J. *et al.* A feedback regulatory loop involving p53/miR-200 and growth hormone endocrine axis controls embryo size of zebrafish. *Sci. Rep.* **5**, 15906; doi: 10.1038/srep15906 (2015).

## Supplementary Material

Supplementary Information
