# Supplementary material for: A feedback regulatory loop involving p53/miR-200 and growth hormone endocrine axis controls embryo size of zebrafish
Source: Sci Rep. 2015 Oct 28;5:15906. doi: 10.1038/srep15906 (PMC4623745; doi:10.1038/srep15906)
Supplement: Supplementary Information [file srep15906-s1.doc]

**Supplementary information**

**A feedback regulatory loop involving p53/miR-200 and growth hormone endocrine axis controls embryo size of zebrafish**

Jing Jing, Shuting Xiong, Zhi Li, Junjie Wu, Li Zhou, Jian-Fang Gui, Jie Mei


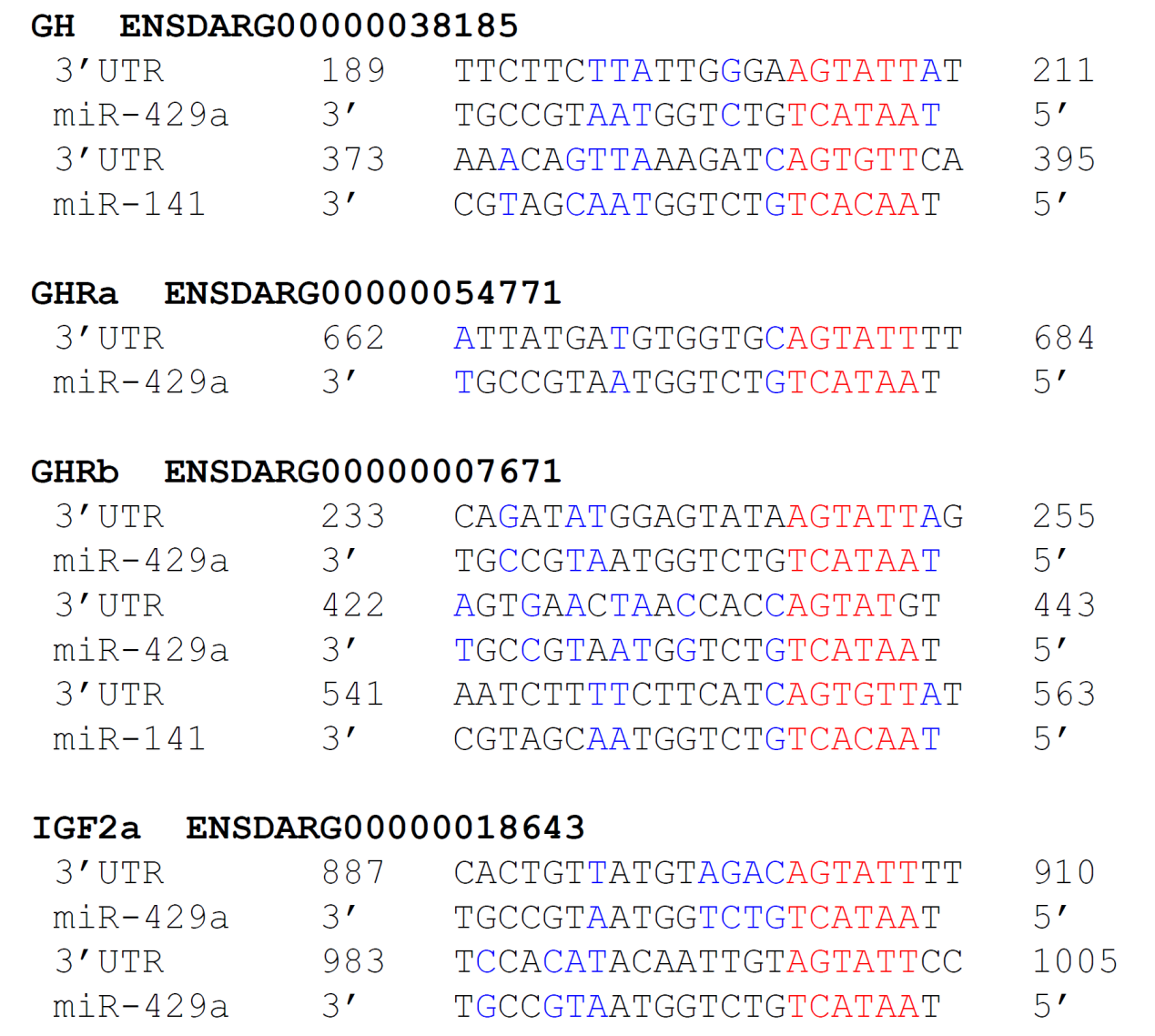


Supplementary Figure 1. Complementarity between miR-200 family members and the predicted target gene in the GH/IGF axis. The predicted target site within the target gene is shown in red; miR-200 seed region is shown in green; other complementarity base is shown in blue.


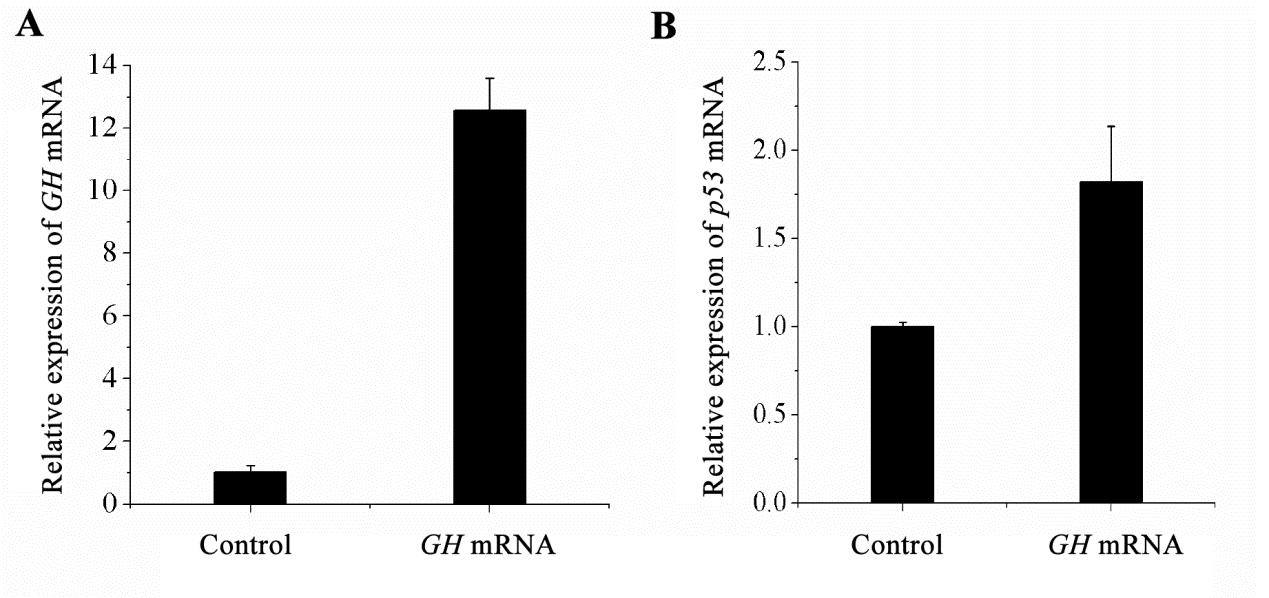


*****

*****

Supplementary Figure 2. The expression level of *GH* (A) and *p53* (B) mRNA in 24hpf embryo after ectopic expression of zebrafish *GH* mRNA. Error bars indicate mean ± SD, n = 3. Student’s t-test was used for statistical analysis (*p < 0.05).

Supplementary Table 1. Primers used for reverse transcription.

| Gene | Stem-loop RT primer |
| --- | --- |
| miR-141 | GTCGTATCCAGTGCAGGGTCCGAGGTATTCGCACTGGATACGACGCATCGT |
| miR-200a | GTCGTATCCAGTGCAGGGTCCGAGGTATTCGCACTGGATACGACACATCGT |
| miR-429a | GTCGTATCCAGTGCAGGGTCCGAGGTATTCGCACTGGATACGACACGGCAT |
| miR-429b | GTCGTATCCAGTGCAGGGTCCGAGGTATTCGCACTGGATACGACATGGCAT |
| miR-200b | GTCGTATCCAGTGCAGGGTCCGAGGTATTCGCACTGGATACGACTCATCAT |
| miR-200c | GTCGTATCCAGTGCAGGGTCCGAGGTATTCGCACTGGATACGACGCATCAT |

Supplementary Table 2. Primers used for qRT-PCR.

| Gene | qRT-PCR forward primer | qRT-PCR reverse primer |
| --- | --- | --- |
| 18s | TCGCTAGTTGGCATCGTTTATG | CGGAGGTTCGAAGACGATCA |
| GH | CCTCTGTCGTTCTGCAACTC | ACTCCCAGGATTCAATGAGG |
| GHRa | ACGAATCTGGACCTCCTACTG | GCAACCCTGAACGACTCACAT |
| GHRb | CCTACAACACAGGGTCAGACT | CTGGGAGTTGTAACTTGGAC |
| IGF1 | CTGGTGCTGTGCGTCCTC | CCACGATGCCACGGTTGT |
| IGF2a | TCCCAGTGTCACAGGCTCT | CTCCATCTGCCTCCTAAAC |
| P53 | TAAGTGATGTGGTGCCTGCCT | TCCTTCGTCCTTCACCATCAG |
| U6 | TGCTCGCTACGGTGGCACA | AGCAATATGGAGCGC TTC |
| miR-141/200a | GCGCTAACACTGTCTGGTAA | GTGCAGGGTCCGAGGT |
| miR-429a | GCGCTAATACTGTCTGGTAA | GTGCAGGGTCCGAGGT |
| miR-200b/c/429b | GCGCTAATACTGCCTGGTAA | GTGCAGGGTCCGAGGT |
